# Using Geographic Information Science to Explore Associations between Air Pollution, Environmental Amenities, and Preterm Births

**DOI:** 10.3934/publichealth.2015.3.469

**Published:** 2015-08-06

**Authors:** Yelena Ogneva-Himmelberger, Tyler Dahlberg, Kristen Kelly, Tiffany A. Moore Simas

**Affiliations:** 1 Department of International Development, Community and Environment, Clark University, Worcester, MA,; 2 Departments of Obstetrics/Gynecology, University of Massachusetts Medical School, Worcester, MA,; 3 Departments of Obstetrics/Gynecology and Pediatrics, University of Massachusetts Medical School/UMass Memorial Health Care, Worcester, MA

**Keywords:** preterm birth, race, Risk Screening Environmental Indicators Model, air pollution, environmental amenities

## Abstract

The study uses geographic information science (GIS) and statistics to find out if there are statistical differences between full term and preterm births to non-Hispanic white, non-Hispanic Black, and Hispanic mothers in their exposure to air pollution and access to environmental amenities (green space and vendors of healthy food) in the second largest city in New England, Worcester, Massachusetts. Proximity to a Toxic Release Inventory site has a statistically significant effect on preterm birth regardless of race. The air-pollution hazard score from the Risk Screening Environmental Indicators Model is also a statistically significant factor when preterm births are categorized into three groups based on the degree of prematurity. Proximity to green space and to a healthy food vendor did not have an effect on preterm births. The study also used cluster analysis and found statistically significant spatial clusters of high preterm birth volume for non-Hispanic white, non-Hispanic Black, and Hispanic mothers.

## 1. Introduction

Each year in the United States about a half million, or 1 in 8, babies are delivered to their mothers preterm, or earlier than thirty-seven weeks gestational age. Preterm birth (PTB) shortens in utero fetal development and has been linked to numerous adverse health outcomes including learning disabilities, respiratory problems, visual issues hearing loss, as well as immediate hospitalization following birth and others [1]. PTB is also a risk factor for low birth weight (LBW, < 2,500 grams) which affects about 316,000 babies born each year in the U.S. [2]. Both PTB and LBW babies are also associated with increased infant mortality, and have higher risks of delayed motor and social skills, as well as learning disabilities [3],[4].

Biologic associations and risk factors for PTB are well documented, and many studies have been performed to test correlations between social and environmental stressors potentially leading to PTB mechanisms, but the exact etiology of PTB is unknown. Biology of stress theories have led scientists to focus on the social determinants that contribute to the incidence of PTB. These can be divided into the two categories: socioeconomic status and environmental exposure. Mothers living in poverty can experience a number of related socio-environmental stressors that may increase the risk of delivering babies preterm or at LBW [5]. For example, poor-quality housing and racially isolated and poor neighborhoods have been demonstrated to increase the likelihood of PTB and LBW [6]–[8]. Race also has an effect on preterm births in the US. According to the National Center for Health Statistics, 16.54% of births to black mothers were preterm, while only 9.68% of births to non-Hispanic white mothers were preterm [9]. This disparity exists with LBW as well, with 11.84% of births to non-Hispanic Black mothers classified as LBW, compared to 5.36% of births to non-Hispanic white mothers.

Environmental stressors, such as air pollution, landfills, and industrial polluting sites, have been linked to PTB and LBW as well. A literature review found that living in proximity to the aforementioned areas confers an increased risk of adverse health outcomes in general [10], and traffic-related air pollution in particular has been shown to increase the risk of adverse birth outcomes [11]–[18].

It is possible that some of the effects of pollution exposure will be mitigated by proximity and access to green space. For instance, an increase in birth weight was associated with an increase in distance to major highways and increase in percent open space [19]. People that live near or have access to green spaces are also more likely to use and derive health benefits from them as compared to people who don't live near them, and the literature suggests a positive relationship between green spaces and an improvement in the health of potential mothers [20]. Tree cover may reduce maternal stress, which might explain why a 10% increase in tree cover within fifty meters of homes was shown to reduce the number of LBW births by 1.42 per 1000 in Portland, Oregon [21]. Another factor that can contribute to maternal health but has not been widely studied is access to healthy foods, which has been shown to affect diet quality during pregnancy. Specifically, women living more than four miles from a supermarket had twice the odds of falling into the lowest level of the diet-quality index for pregnancy versus women located within four miles [22].

Most studies on adverse birth outcomes that use GIS technology are usually limited to mapping maternal addresses and calculating their distances to polluting facilities or major roads. Studies that use methods of spatial cluster analysis and mapping to explore the spatial distribution and pattern of adverse birth outcomes are still limited [23]–[25].The identification of “hot spots” or clusters of certain events (areas with high density of PTB, for example) is an important investigative first step as it allows researchers to focus further on these specific areas and to develop a targeted intervention strategy [26]. The first objective of our study is to analyze the spatial distribution of preterm births for three racial groups (non-Hispanic Blacks, Hispanic and non-Hispanic white) in Worcester, MA to determine if they are clustered or randomly distributed in the city. Our specific research question is: Are there spatial clusters on preterm births in Worcester? Statistically significant spatial clusters would suggest that there is some underlying process or characteristic of space that could be associated with these clusters.

Multiple studies have concluded that environmental factors are important contributors to birth outcomes, along with social and host factors [27]. While some of these environmental factors have been studied extensively in the context of the adverse birth outcomes (air pollution), others have not. Our study attempts to bring those less and more studied environmental factors together and use spatial statistics to explore their potential effects on PTB. More specifically, our second objective is to analyze associations between PTB and distance to major roads, exposure to hazardous air pollutants from stationary sources, access to vendors of healthy food, and access to green space and parks. In other words, the study addresses the following research question: are there statistical differences between full term and preterm births in their access to environmental amenities (green space and vendors of healthy foods) and exposure to air pollution?

## 2. Materials and Method

### 2.1. Study area

Worcester's population of 182,669 makes it the second-largest city in New England after Boston [28]. Worcester has a long history that dates back to its incorporation as a town in 1722 [29]. Manufacturing was a strong driver of Worcester's economy throughout the industrial revolution and into the 1950s, after which manufacturing declined and the city lost 20% of its population over the next thirty years [30].

As a former manufacturing base Worcester is home to a number of brownfields, or abandoned but desirable building sites with unknown levels of pollutants that are part of the EPA's National Priorities List [31]. There are also a number of actively polluting TRI sites monitored by the EPA that are located near population centers within and around the city [32]. Multiple state and Federal highways pass through the city, carrying some 270,000 pollution-emitting automobiles every day [33].

Worcester is also home to dense “triple-decker” housing, living units built during the industrial era that typically house a family on each floor [34]. Dense triple-decker housing and wave after wave of immigrants, including Italian, Swedish, Lithuanian, Jewish, sub-Saharan African, Polish, Syrian, and Vietnamese among many others has resulted in a 40% minority city with a population density of 4,678 persons per square mile. Its poverty rate is 20%, twice the Massachusetts state average. The high school graduation rate in Worcester is 5% lower than the state average, and the percent of people with four-year bachelor's degrees is 10% lower than the state average [28],[35]. Worcester's confluence of dense, low-income populations concentrated near highways, TRI sites, and brownfields is an interesting setting to investigate the social and environmental effects of this kind of living situation on preterm birth and low birth weight babies.

### 2.2. Data

Birth data for residents of the city of Worcester delivered between 4/1/2006 and 3/31/2011 were provided by UMASS Memorial Health Care (7377 records total) after approval by the Institutional Review Board of its academic partner, the University of Massachusetts Medical School and the Institutional Review Board of Clark University. UMASS Memorial Health Care delivers on average about 50 percent of all births in Worcester County (central Massachusetts).

The data included mother's residential address and race/ethnicity, baby's birth date, gender, estimated gestational age (EGA) in weeks, birth weight, singleton or multiple birth, and if it was a live birth. We used the address database from the GIS Office of Worcester to geocode and map birth data. This database consists of about 48,000 points corresponding to individual buildings and allows identifying each birth's location with a high degree of spatial accuracy. The geocoding success rate was 99.8% (only 16 addresses of 7377 were unmatched). We selected only singleton births (7217 records), and from them further selected only live births (7136 records). We then separated singleton live births into two groups for each race/ethnicity—full term (≥ 37 weeks EGA) and preterm (<37 weeks EGA). There were 613 PTB total (see Table 1). It should be noted that percent preterm births reported in this table is lower than typically cited as national average ( i.e. 11-12%) as we considered singletons only.

To examine PTB in more details we divided them into three groups based on the degree of prematurity: late preterm (EGA = 34-<37 weeks; 430 births); early preterm (EGA = 28-<34 weeks; 158 births), and extremely preterm (EGA < 28 weeks; 76 births). The thresholds for the groups were selected based on what has been reported in the literature [36].

**Table 1.** Prevalence of preterm birth in the cohort by race (n = 7136).

Race	Total births	Preterm births	% preterm births
Non-Hispanic Black	1376	136	9.89
Non-Hispanic White	3209	265	8.25
Hispanic	2564	212	8.26

To explore socio-economic context of PTB we used Environmental Justice map obtained from MassGIS [34]. Environmental justice (EJ) areas are defined as neighborhoods that meet one or more of the following criteria: >25% of residents are minorities, median household income is ≤65% of the statewide median, and English language isolation present in ≥25% of residents. These calculations use Census 2010 blockgroup data and the American Community Survey (ACS) 2006–2010 five-year estimates tables. Historically, these communities have been disproportionately impacted by environmental hazards and the lack of environmental assets, such as urban parks and green space. In Worcester, 70.7% population lives in EJ areas [37].

**Figure 1.**
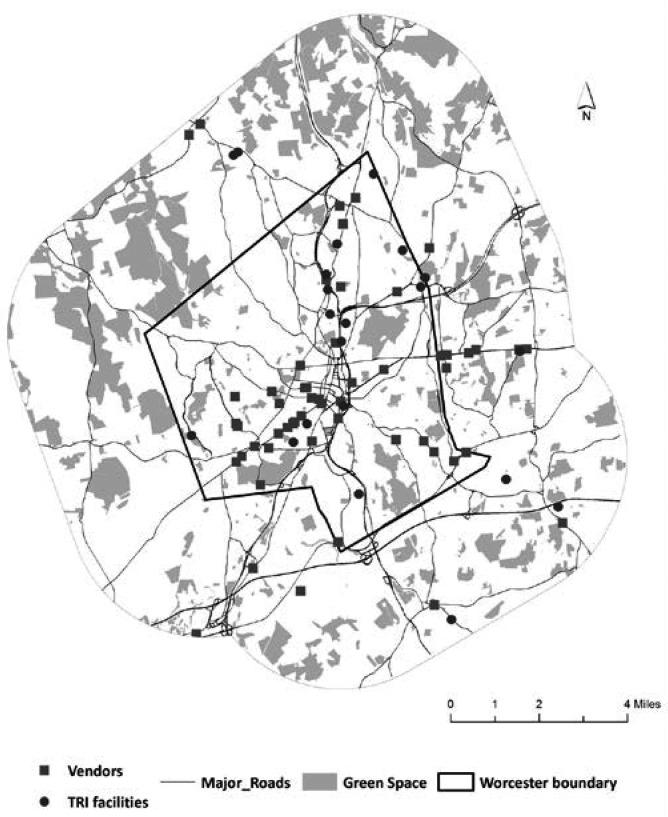
TRI facilities, major roads, vendors of healthy food,and green space in Worcester and its vicinity.

All environmental variables were obtained from public sources. We downloaded relevant data not only for the City of Worcester, but also for the surrounding areas within 5 km of the City. This allowed accounting for nearest points of interest (vendors, parks, and polluting facilities) that are outside the city border to be included in the calculations of distances (Figure 1).

Distance to nearest major roads for each birth was calculated using Massachusetts Department of Transportation roads data available from MassGIS [38]. The following four road categories were considered major roads according to Mass DOT: limited access highway; multi-lane highway, not limited access; other numbered route; and major road—arterials and collectors. Distance was calculated as the shortest straight line distance using spatial join algorithm in GIS.

To find vendors of healthy foods we used Reference USA (http://www.referenceusa.com/), a commercial database of all businesses in the country, to which we had access as patrons of Worcester public library. We selected the following vendor types based on the North American Industry Classification System (NAICS) codes: supermarkets and other grocery stores, except convenience stores (code 445110), fruit and vegetable market (code 445230), department stores except discount stores (code 45211101) and warehouse clubs and supermarkets (code 45291001). These categories include large chain supermarket such as Stop and Shop, Shaw's, Price Chopper, smaller non-chain ethnic supermarkets, such as Compare Foods, small markets that exclusively sell fruits and vegetables, and department and warehouse stores, such as Wal-Mart Supercenter, Sam's Club, and BJ's Club. There were a total of 59 vendors in Worcester and the surrounding towns; their addresses were geocoded using the Worcester address database.

Green spaces and parks data for Worcester and surrounding towns were obtained from Protected and recreational open space layer available from MassGIS [39]. This layer contains parks, playgrounds, recreational complexes, wildlife sanctuaries and other conservation areas where residents can walk, play, and exercise. Distance from each birth to the nearest vendor and to the green space was calculated using network analysis tools in ArcGIS Desktop. Street data from the City of Worcester were used to build the network.

The main source of air pollution data are regional monitoring stations that provide continuous measurements over 24-hour periods on a variety of air pollutants, most commonly including Ozone, Carbon Monoxide, Sulfur Dioxide, Nitric Oxide, Nitrogen Dioxide, Total Reactive Oxides of Nitrogen, Lead, Particulate Matter or PM10 (10 microns & smaller), and Fine Particles or PM2.5 (2.5 microns & smaller). Unfortunately, stations are often located far from each other, so their data is most suitable for a regional level assessment rather than individual-level exposure. For example, there are only three air-monitoring stations in Worcester, MA making the data unsuitable for individual-level exposure analyses. An alternative approach to modeling air pollution exposure is to use emissions data from polluting facilities. For example, the Environmental Protection Agency (EPA) collects data for the Toxic Release Inventory (TRI) every year from more than 20,000 U.S. industrial facilities [32], and reports emissions data not only for each chemical separately, but also for groups of chemicals (hazardous air pollutants, carcinogens, metals and metal compounds, persistent bioaccumulative, and toxic chemicals). The data includes geographic coordinates of the facility, and the amount of releases per year (in pounds) for each chemical and holds a lot of potential for local level air pollution exposure studies, but so far has been underutilized in research on adverse birth outcomes. For this study, we selected hazardous air pollutants (HAP) because of their links with LBW and PTB. There are currently 190 different chemicals on the HAP list (http://iaspub.epa.gov/triexplorer/tri_text.list_chemical_hap). We downloaded data for 2005-2010 and linked each birth to the corresponding year of the TRI release. For babies born before April 15 of each year we used TRI data from the previous year to account for the exposure during the first half of the pregnancy. The number of TRI facilities that reported HAP releases ranged between 64 and 96 for the six years we examined.

We used two approaches to model maternal exposure to these pollutants. First, we calculated straight-line distance to the nearest TRI facility for each birth using spatial join in GIS. The second approach used a modeled hazard score and was based on the amount of release and its toxicity. The data for the second approach came from the EPA's Risk-Screening Environmental Indicators (RSEI) Model [40]. This model takes into account fate and transport of pollutants, pounds of release, and chemical-specific toxicity weights and produces “modeled hazard score” and other measures for each TRI facility. The higher the score the more hazardous is the facility to human health. Detailed information about the model can be found in the RSEI Methodology paper [40]. Modeled hazard scores were used to study unequal exposure to hazardous industrial facilities in Philadelphia [41] and Hillsborough County in Florida [42]. In order to link these scores to each birth, we created a continuous surface of modeled hazard scores using a kernel density function in GIS (search radius of 1500 ft and 20 ft x 20ft output pixel size) and extracted the modeled hazard score from the underlying raster pixel for each birth. Figure 1 shows locations of TRI facilities, vendors of healthy food, major roads and green space.

### 2.3. Methods

As the first step, to visually represent the spatial distribution of PTB we created kernel density maps for each race separately. This technique shows general patterns of distribution while masking the exact location of the points and thus preserving confidentiality of the data. We used Spatial Analyst in ArcGIS 10.1 with a quadratic kernel function [43] and search radius of 1500 ft. The output raster surfaces show the density of PTB near each 30 ft x 30ft raster cell. In the next step we used a variety of analytical methods, including spatial cluster analysis and analysis of variance.

#### 2.3.1. Spatial clustering

While most research on birth outcomes uses birth data aggregated to census block group, tract, city block, town, zip code or county, some researchers used mother's residence address and geocoding tools to create maps depicting locations of births. These studies then used point locations on the map as inputs for distance and buffer operations in GIS to calculate exposure to air pollution from traffic [44]–[48] or landfills [49]. However, no studies have used GIS and methods of spatial statistics to identify spatial clusters of adverse birth outcomes. These methods enabled us to answer the following questions: Are pre-term births distributed randomly within the city or are they spatially clustered. If they are clustered, where are these clusters located?

To answer these questions we used two spatial clustering methods—Nearest Neighbor Hierarchical (NNH) Clustering and Risk-Adjusted Nearest Neighbor Hierarchical (RNNH) Clustering in CrimeStat software [50] to find statistically significant spatial clusters of PTB for each racial group. Both methods identify spatial groupings of observations that are closer to each other than would be expected if the distribution was completely random, however there is an important difference between them. While the former method identifies clusters representing high volume of events (PTB), the latter method takes into account the distribution of the underlying population (i.e., locations of all births) and finds areas where PTB points are closer than would be expected from the underlying population. In criminology, the RNNH approach is used to analyze spatial distribution of one type of crime, e.g., robberies, in relationship to the overall distribution of crimes. In this case, all crimes are used as the baseline population and the output represent clusters of high risk of robberies [50]. In our study, RNNH identifies areas of high risk of PTB given distribution of all births. These approaches have been used traditionally in crime analysis, including identifying drug markets [51], as well as in other research areas, such as clustering of mobile produce vendors in Bronx, NY [52] and clustering of falls in Hong Kong [53].

The NNH technique uses two criteria for grouping points into clusters—the threshold distance and the minimum number of points per cluster. The threshold distance is related to the probability level for selecting any two points as a pair by chance. The smaller the threshold distance, the lower is the probability of identifying a cluster by chance (for more details see chapter 7 in [50]). The second criterion is the minimum number of points necessary for a cluster. The user arbitrarily sets both criteria, and it is recommended that the user start with the default settings and experiments with several runs “to get a solution that appears right” [50, page 7.23]. We have used a threshold distance corresponding to 5% probability of identifying a cluster by chance (most commonly used setting) and changed minimum number of points per cluster between 3 and 10 to test sensitivity of results.

The RNNH technique uses one additional parameter—the distribution of the underlying population in a form of a kernel density surface. This continuous surface is interpolated from the point data of all births within each racial/ethnic group and shows areas of high and low birth concentrations. The algorithm uses this information to dynamically adjust the threshold clustering distance according to the varying densities of births. In other words, in areas with high birth density, the threshold distance is shorter, and in areas with low birth density, it is larger. There are several options for the interpolation method (density functions) and the choice of kernel bandwidth (fixed distance or adaptive). We used a normal density function (most commonly used setting) and an adaptive bandwidth with a minimum of 25 points.

Monte-Carlo simulation was used to test statistical significance of clusters produced by each NNH and RNHH run. This simulation assigns the same number of observations to random locations within a rectangular area of the same size as the study area (38.44 sq. miles for Worcester), and evaluates the number of clusters according to the parameters previously set by the researcher. The simulation is repeated multiple times, and for each simulation, the number of clusters generated from a random distribution is compared to the number of observed clusters.

#### 2.3.2. Analysis of associations with air pollution and environmental amenities

To analyze associations with environmental factors we first ran a t-test to compare the means for the two groups (PTB and full-term) for all races together for the following variables: distance to major roads, distance to vendors of healthy foods, distance to the nearest green space, distance to the nearest TRI facility, and modeled hazard score for RSEI. Then we repeated the analysis for each race separately. We used Welch's t-test because of unequal variances within the samples. We also used ANOVA to compare the means of these variables for three PTB groups based on the degree of prematurity. All tests were done in SPSS software (version 20.0) and statistical significance of the tests was assessed at p = 0.05.

## 3. Results

### 3.1. Spatial Clustering

Table 2 summarizes the results of NNH and RNNH clustering. For each race, we started with the default setting of 10 points per cluster and lowered it until at least one cluster formed. Monte-Carlo simulation was also run concurrently to have a measure of statistical significance of these clusters. For most iterations of spatial randomization, zero clusters were produced, but for some—only one spatial cluster was produced. For the final result we chose the runs that produced the largest number of clusters from the observed data and zero clusters by chance. Spatial clusters are represented by ellipses which correspond to one standard deviation from the mean center of the cluster [47]. We report results by race below.

**Figure 2.**
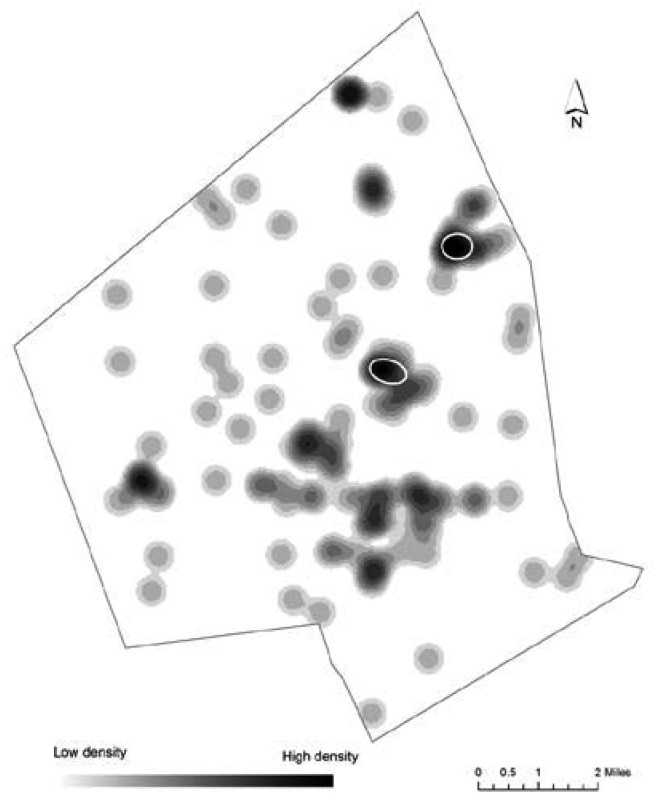
Density of PTB for non-Hispanic Black mothers and statistically significant NNH clusters.

There were 136 PTB to non-Hispanic Black mothers, and NNH clustering produced two spatial clusters of high volume of PTB: one cluster with 12 and the other with 9 PTBs. Figure 2 shows PTB density for non-Hispanic Black mothers with two clusters overlaid. Of the 21 PTBs in clusters, seven are located in the areas designated as environmental justice areas due to low income level and high minority population, and 14 are located within EJ areas that received this designation due to high percent of low income, minority and linguistically isolated people. RNNH clustering produced one spatial cluster of high PTB risk area. Interestingly, this cluster is different from the two clusters identified by the NNH routine, and contains five PTBs located at the same address. Close examination of the data reveals that two of the five births were to the same mother; and two were also extremely preterm. Through web search we discovered that an affordable housing apartment complex is located at this address.

**Table 2.** Sensitivity analysis for NNH and RNNH. Numbers in bold correspond to settings that produced the highest number of statistically significant clusters.

Min number of points per cluster	Number of clusters identified	Clusters obtained by chance (95%)
**Non-Hispanic Black-NNH**		
10	1	0
9	1	0
8	2	0
**7**	**2**	**0**
6	3	1
**Non-Hispanic Black-RNNH**		
4	1	0
**3**	**1**	**0**
**Non-Hispanic White-NNH**		
10	1	0
9	1	0
8	3	0
**7**	**4**	**0**
6	5	1
**Non-Hispanic White-RNNH: ** No clusters found		
**Hispanic-NNH**		
7	4	0
**6**	**8**	**0**
5	9	1
**Hispanic-RNNH: ** No clusters found		

**Figure 3.**
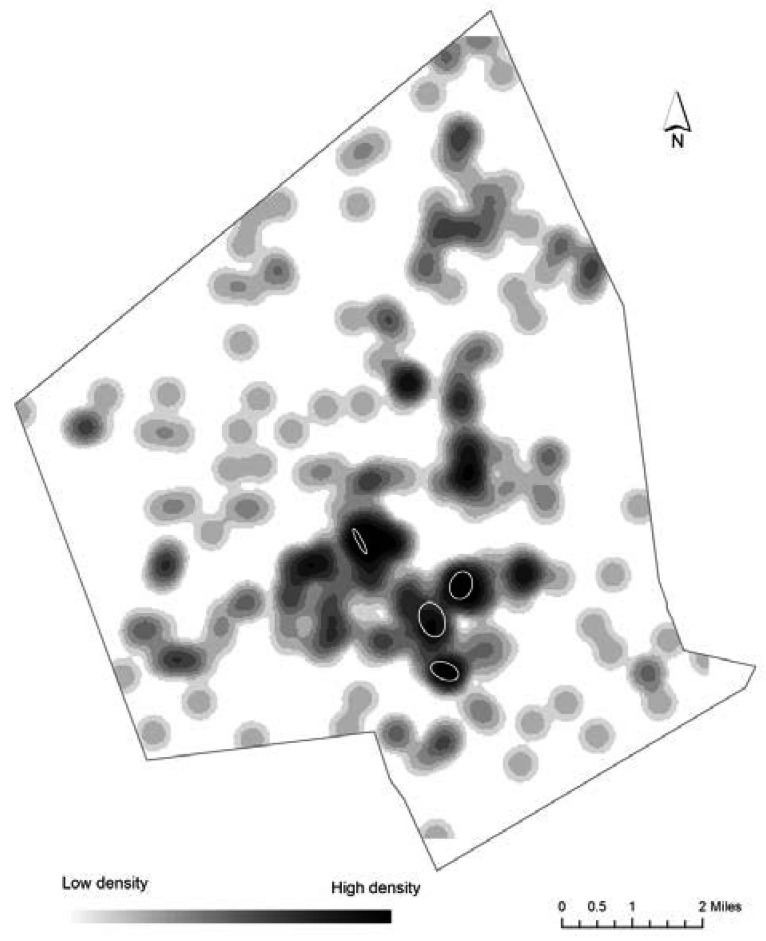
Density of PTB for non-Hispanic white mothers and statistically significant NNH clusters.

There were 265 preterm births to non-Hispanic white mothers, 37 of them formed 4 spatial NNH clusters, containing between 8 and 11 births each (Figure 3). All of these clusters were inside EJ areas. More specifically, 11 births were located in high minority EJ areas, 12 in the low income and high minority EJ areas, and 14 births within areas that satisfy all three EJ criteria (high poverty, high level of linguistic isolation and high minority). When RNNH technique was applied, no spatial clusters of high risk were produced.

**Figure 4.**
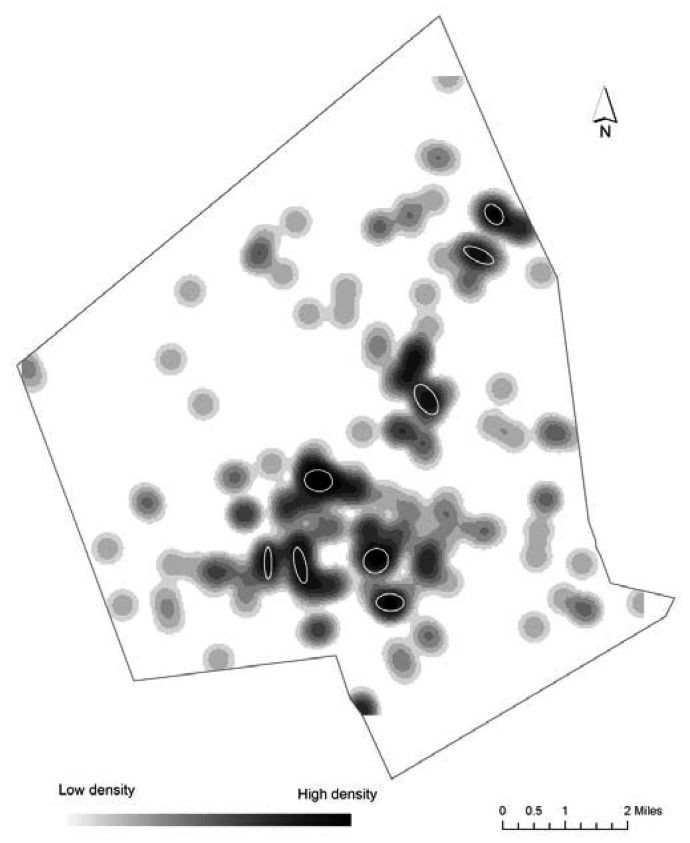
Density of PTB for Hispanic mothers and statistically significant NNH clusters.

There were 212 PTB to Hispanics mothers, and 71 of those formed 9 spatial clusters, with 7 to 13 PTB each (Figure 4). As with non-Hispanic white PTBs, all of these spatial clusters fell inside the EJ areas. Six births are in minority EJ areas, 25 in high minority and low income areas, and 40 births are from the EJ areas with high poverty, high levels of linguistic isolation and high minority populations. When RNNH technique was applied, no spatial clusters of high risk were produced.

### 3.2. Analysis of associations with air pollution and environmental amenities

Table 3 summarizes the group means and t-test significance for the five variables used in the analysis. When all births, regardless of maternal race, are taken together, the t-test shows that the mean distance to the nearest polluting site for the preterm births is significantly (p < 0.05) smaller than for the full term births (4272 ft vs. 4549 ft). When births to non-Hispanic Black mothers are analyzed separately, the t-test shows that preterm births are exposed more to air pollution, as the hazard score for this group is significantly (p < 0.05) higher than for the full term births (5.22E + 07 vs. 1.57E + 07). No significant differences were observed in the mean values for any variables between preterm and full term births for non-Hispanic white and Hispanic mothers.

A closer examination of the preterm births stratified into three groups by EGA reveals (Table 4) that the air pollution exposure is significantly higher (p < 0.05) for the extremely preterm births (EGA < 28 weeks) in comparison with the other two preterm groups: the mean hazard score for these births is at least 5 times higher (1.19E + 08 versus 2.17E + 07). The same pattern holds true when three preterm groups are analyzed separately for the non-Hispanic white and Hispanic mothers – the hazards score for the most extreme preterm group in non-Hispanic white mothers is 5.6 time higher than for the least preterm group (1.37E + 08 versus 2.44E + 07); it is almost 13 times higher for Hispanic mothers (1.01E + 08versus 7.81E + 06). No other statistically significant differences were observed for the other four spatial variables.

**Table 3.** Comparison of means for environmental factors for preterm and full-term births by race. All distances are measured in feet. Statistically significant values (p < 0.05) are marked with an asterisk.

	Distance to healthy food	Distance to green space	Distance to major road	Distance to TRI facility	TRI modeled hazard score
All births					
FT	3857	1978	729	4549	2.23E + 07
PT	3819	1930	704	4272	3.24E + 07
T-test	0.323	1.113	0.977	2.387*	-1.099
Non-Hispanic Black					
FT	3888	2025	740	4676	1.57E + 07
PT	3760	1956	737	4409	5.22E + 07
T-test	0.530	0.683	0.060	1.082	-2.407*
Non-Hispanic White					
FT	4076	2033	744	4769	2.77E + 07
PT	4004	1952	684	4428	3.48E + 07
T-test	0.368	1.204	1.638	1.905	-0.574
Hispanic					
FT	3567	1885	706	4207	1.90E + 07
PT	3625	1887	710	3990	1.67E + 07
T-test	-0.312	-0.024	-0.093	1.116	0.189

**Table 4.** Comparison of means for environmental factors for three groups of preterm births by race. All distances are measured in feet. Number of cases in each group is in parenthesis. Statistically significant values (p < 0.05) are marked with an asterisk.

	Distance to healthy food	Distance to green space	Distance to major road	Distance to TRI facility	TRI modeled hazard score
**All births**					
Moderate PT (430)	3799	1885	692	4325	2.17E + 07
Very PT (158)	3802	1916	700	4178	1.17E + 07
Extremely PT (76)	3799	2048	760	4080	1.19E + 08
F-statistic	0.000	0.844	0.432	0.359	7.632*
**Non-Hispanic Black**					
Moderate PT (81)	3933	1932	675	4631	4.37E + 07
Very PT (40)	3420	1753	675	3786	2.90E + 06
Extremely PT (30)	3634	2184	830	4531	1.16E + 08
F-statistic	.602	1.323	.620	1.411	.960
**Non-Hispanic White**					
Moderate PT (185)	4039	1974	696	4524	2.44E + 07
Very PT (69)	3870	1899	685	4447	2.31E + 07
Extremely PT (25)	4114	1928	765	3820	1.37E + 08
F-statistic	.091	.134	.194	.711	4.207*
**Hispanic**					
Moderate PT (164)	3464	1761	695	3948	7.81E + 06
Very PT (49)	4020	2071	741	4119	2.99E + 06
Extremely PT (21)	3662	1997	655	3746	1.01E + 08
F-statistic	.855	2.617	.200	.155	7.033*

## 4. Discussion and Conclusions

We found statistically significant clusters of high PTB volume for all three racial/ethnic groups. They represent high concentrations of incidents that did not happen due to a random chance. All of these clusters were located in areas designated as environmental justice areas, meaning that people who live in these locations are more vulnerable to potential exposure to harmful effects of environmental pollution. When overlaid on top of the PTB density maps (Figures 2–4) these clusters cover areas with the highest density of PTB, thus illustrating how complimentary the two approaches are to each other for visual representation of point patterns.

When risk-adjusted NNH routine was applied to the data, only one cluster of high PTB risk among non-Hispanic Black women was found, with all PTBs located at one particular address. Further exploration of our data in GIS revealed that this cluster is located in close proximity to the top air polluter in Worcester (largest number of pounds of HAP released each year between 2006 and 2010). This facility, KT Acquisition LLC, specializes in iron and steel forging and emits chromium compounds, cobalt compounds, lead and nickel compounds. According to the EPA, “exposure to chromium (VI) may result in complications during pregnancy and childbirth [51].

Our results are consistent with the findings of previous studies that industrial air pollution exposure has an effect on PTB. We modeled exposure to air pollution using two different measures—proximity of the polluting facility and its modeled hazard score. The first measure was significant when all PTB were compared to full term births. This indicates that proximity to TRI sites has an effect on PTB outcome regardless of race. When PTB were analyzed by maternal race, the second measure proved significant for non-Hispanic Black PTB as compared to full term non-Hispanic Black births. The hazard score was also a statistically significant factor when PTB were split into three groups based on the degree of prematurity: the hazard score was much higher for the extremely preterm group, both when PTB were considered all together (regardless of maternal race) and when split by race (for Hispanic and non-Hispanic white mothers only).

A major strength of this study was the use of the novel air pollution data set from RSEI. A majority of the birth outcomes studies use government monitoring station data to assess exposure. However, dense networks of air pollution monitors exist only in large urban centers, and medium-size cities like Worcester do not have enough stations to be useful in spatial analysis. RSEI data provides several advantages. First, it combines source-specific data on release amount, stacks height and exit gas velocities and uses a fate-and-transport model to estimate ambient concentrations around each facility. Second, RSEI weighs chemicals by their toxicity thus improving the measure of the potential health effect due to air pollution. Additional studies utilizing source-specific information on pollutants, such as RSEI, could provide further evidence on pollutants impact on pregnancy outcomes and may help inform public health policy decisions.

Another study strength is that exposure to air pollution and access to environmental amenities were estimated at the individual level. Very few studies examine the relationship between birth outcomes and “positive” elements of the environment, and our study attempted to address this gap in the literature. However, we found no association between proximity to major roads, proximity to healthy food vendors, and proximity to green space and PTB. Our findings illustrate that even sophisticated distance calculation methods (street network-based vs. a straight line) have difficulty explaining the variation of birth outcomes because other important individual-level factors are not included in the analysis.

The unavailability of individual maternal data on behavior (tobacco use, drugs, nutrition, and physical activity level), health issues (hypertension, diabetes, uterine abnormalities, infections, etc.), socio-demographic factors (age, place of birth, access to prenatal care, and socioeconomic status), psychological factors (level of stress, social support network) and neighborhood conditions is the major limitation of our study. All of these factors play an important role in pregnancy outcomes, including PTB, but they were not available to us.

A second limitation is that the birth data we used accounts for about a half of all births in the city, thus leading to a potential selection bias, smaller sample size and small clusters of outcomes. Our findings could have been different if we had access to data on all births in the city. There are several reasons why we chose to use birth data from the UMass Memorial Health Care (UMMHC). Of relevance, the impetus to this study was a noted public health trend of significant disparities in infant mortality rates to black and Hispanic residents of the city in which UMMHC is located (Worcester, MA). This notable difference was especially prominent regarding mortality at peri-viable gestations and thus prompted our focus on preterm birth. UMMHC is a tertiary care referral center for the region and thus we were confident that these high risk gestations and early deliveries would be captured utilizing our health system data. This study focused on piloting and refining spatial clustering methods prompted by this public health issue and hence we used readily available records that we knew would capture the deliveries of greatest interest. Future studies would seek to validate these methods in larger datasets and would require access to birth certificate data for the entire city.

A third limitation is related to the fact that we based our assessment on self-reported maternal addresses; we assumed that the mothers lived at the reported birth address during the entire pregnancy and that their air pollution exposure was constant. Their exposure to pollution could have been different at earlier stages of pregnancy if they moved to this address soon before the birth. In the study conducted by Brauer et al. [12] 35 % of the population changed residency during pregnancy, so future exposure modeling studies should take this factor into consideration.

An additional limitation is related to the uncertainty associated with the modeled hazard score, due to the subjective nature of the parameters in the kernel density technique. While we acknowledge that the resulting raster density surface may look somewhat different if the input parameters were modified, we are confident that the surface we used represents the general spatial pattern of the hazards score accurately.

Despite the limitations discussed above, we believe that our study provides useful results and makes a unique contribution to the existing body of literature. It is the first study to apply two hierarchical nearest neighbor clustering techniques in the context of birth outcomes. Our findings illustrate the usefulness of these two techniques and their complimentary nature. When used in concert they allow the identification of two characteristics of spatial distribution—the high volume and high-risk areas of PTB. We hope that in the future these approaches are used more in public health studies in general, and in birth outcome studies in particular. Furthermore, our study is methodologically unique because it used RSEI to estimate the potential hazard from exposure to industrial air pollution. RSEI is a complex and rich model that has been peer-reviewed [40] but so far has not been used broadly in the scientific community.

GIS and spatial statistics are useful tools for studying birth outcomes. The study's result indicated that there are statistically significant spatial clusters of preterm births in Worcester for non-Hispanic Black, non-Hispanic white and Hispanic mothers. These clusters were located in areas with low income and/or a high percent of linguistically isolated population. Preterm births are located closer to polluting facilities and have higher hazard score than full term births. However, there was no evidence of associations between PTB and proximity to the nearest major road, to parks and to vendors of healthy foods. Information about traffic density and individual maternal nutritional and exercise habits would be necessary to make these associations more meaningful.
